# The state of the art of adeno-associated virus-based vectors in gene therapy

**DOI:** 10.1186/1743-422X-4-99

**Published:** 2007-10-16

**Authors:** Renata dos Santos Coura, Nance Beyer Nardi

**Affiliations:** 1 Department of Genetics, Universidade Federal do Rio Grande do Sul, Av Bento Goncalves 9500, 91501-970, Porto Alegre, RS, Brazil

## Abstract

The adeno-associated virus (AAV) has rapidly gained popularity in gene therapy since the establishment of the first AAV2 infectious clone, in 1982, due to some of their distinguishing characteristics such as lack of pathogenicity, wide range of infectivity, and ability to establish long-term transgene expression. Notably over the past decade, this virus has attracted considerable interest as a gene therapy vector, and about 85% of the currently available 2,041 PubMed references on adeno-associated viruses have been published during this time. The exponential progress of AAV-based vectors has been made possible by the advances in the knowledge of the virology and biology of this virus, which allows great improvement in AAV vectors construction and a better comprehension of their operation. Moreover, with the recent discovery of novel AAV serotypes, there is virtually one preferred serotype for nearly every organ or tissue to target. Thus, AAV-based vectors have been successfully overcoming the main gene therapy challenges such as transgene maintenance, safety and host immune response, and meeting the desirable vector system features of high level of safety combined with clinical efficacy and versatility in terms of potential applications. Consequently, AAV is increasingly becoming the vector of choice for a wide range of gene therapy approaches. This report will highlight the state of the art of AAV-based vectors studies and the advances on the use of AAV vectors for several gene therapy approaches.

## Background

The adeno-associated virus (AAV) is a small, icosaedral and nonenveloped virus that belongs to the parvovirus family, specifically the Dependovirus genus. The members of this genus require a helper virus, such as adenovirus or herpes simplex virus, to facilitate productive infection and replication. In the absence of a helper virus, AAVs establish a latent infection within the cell, either by site-specific integration into the host genome or by persisting in episomal forms. The wild AAV capsid has approximately 22 nm and encapsidates a linear single-stranded DNA genome of about 4.7 kb of either plus or minus polarity [1,2]. The AAV2 DNA termini consists of a 145 nucleotide-long inverted terminal repeat (ITR) that forms a characteristic T-shaped hairpin structure, due to the multipalindromic nature of its terminal 125 bases, which allows its fold on itself via complementary base pairing [3], forming a secondary structure that provides a free 3' hydroxyl group for the initiation of viral DNA replication [4]. This viral replication process relies on host cell polymerase activities, since AAV does not encode its own polymerase [5]. These ITRs are the only cis-acting elements required for genome replication and packaging, and flank the two large open reading frames (ORFs) of the virus genome. The left ORF, Rep (replication), encodes four replication proteins (Rep 78, Rep 68, Rep 52, and Rep 48), through the use of two different promoters and alternative splicing, responsible for site-specific integration, nicking, and helicase activity, as well as regulation of promoters within the AAV genome. The right ORF, Cap (capsid), encodes, through alternative mRNA splicing and alternative start codon usage, the three viral structural proteins (VP1, VP2 and VP3) that assemble at a ratio of approximately 1:1:10, respectively, to form a mature AAV particle [6].

Following the establishment of the first infectious clone of AAV serotype 2 (AAV2) in 1982 [7] and the pioneering work on the successful cloning of AAV establishing the foundation of recombinant AAV vectors capable of expressing foreign genes in mammalian cells [8,9], in the early 1980s, AAV2 vectors have rapidly gained popularity in gene therapy applications, due to some of their distinguishing biological features. The unique life cycle of AAV demonstrates how this class of viruses has adapted to coexist with mammalian hosts in a manner that allows for long-term persistence without any detectable deleterious effect on the host. Despite the deceptively simple structure of AAV, this virus is able to use its nonstructural proteins to facilitate replication as a satellite of other DNA viruses during its productive phase, as well as to establish stable integrated and episomal forms during its latent phase [4,10,11]. This requires numerous complex interactions between AAV genomic elements, AAV proteins, host proteins, and helper virus proteins [12]. Many of those mechanisms have been elucidated in detail for the AAV2, the best characterized AAV serotype [13,14]. Therefore, the nonpathogenic and persistent long-term nature of AAV infection combined with its wide range of infectivity have made this virus an important candidate as a therapeutic gene transfer vector.

Recombinant adeno-associated viral (rAAV) vectors have rapidly advanced to the forefront of gene therapy in the past decade. The exponential progress of AAV-based vectors has been made possible by the advances in the knowledge of the virology and biology of this virus, which allows great improvement in AAV vectors construction and a better comprehension of AAV vectors operation. Moreover, with the recent discovery of novel AAV serotypes, there is virtually now one preferred serotype for nearly every organ or tissue to target, since these isolates are ideally suited to development into human gene therapy vectors due to their diverse tissue tropisms and potential to evade preexisting neutralizing antibodies against the common human AAV serotype 2. Thus, rAAV-based vectors have been successfully overcoming the main gene therapy challenges and meeting the desirable vector system features of high level of safety combined with clinical efficacy and versatility in terms of potential applications. Consequently, rAAV are increasingly becoming the vector of choice for a wide range of gene therapy approaches.

This review will originally highlight the state of the art of AAV-based vector studies and the advances in the use of AAV vectors in several gene therapy approaches.

### Characteristics of AAV-based vectors

As mentioned above, in the early 1980s, pioneering work on the successful cloning of AAV established the foundation of recombinant AAV vectors capable of expressing foreign genes in mammalian cells. Since then, the AAV has been more and more studied and considered for gene therapy applications. In all, 85% of the available (up to June 2007) 2,041 PubMed references on AAV were published during the last 10 years.

This increasing interest on AAV is justified by its characteristic features that distinguish it from many other viral vector systems, such as retro/lentiviral and adenoviral vectors, and turn it into a very attractive tool for gene therapy. These features, as mentioned above, include: (1) its nonpathogenicity and nonimmunogenicity as well as its heat stability and resistance to solvents and to changes in pH and temperature [15]; (2) AAV vectors only retain about 300 nucleotides of viral sequence in the form of nontranscribed ITRs, which greatly improves its safety for human clinical applications by reducing the risk of recombination with wild-type virus. Moreover, lack of viral coding sequences extends the duration of gene expression as no viral gene products are expressed in target cells, which reduces the risk of eliciting a cellular immune response; (3) AAV vectors have a broad host and cell type tropism range and transduce both dividing and nondividing cells in vitro and in vivo. Furthermore, the recent discovery of novel AAV serotypes will expand even more the universe of potential target organs, tissues and cells; and (4) AAV vectors maintain (over several years) high levels of gene expression in vivo, in the absence of a significant immune response to the transgene product. This is a major requirement for gene therapy approaches for some diseases, and constitutes the most promising and distinguishing features of AAV vectors.

However, there are also a few drawbacks in using AAV vectors for gene therapy applications. (1) Their size limits the insertion of gene expression cassettes. Whereas strategies have been developed to overcome this limitation, those approaches still suffer from low efficiency resulting in decreased levels of gene expression. (2) Gene expression is generally of slow onset, due to the requirement of conversion of the single-stranded AAV DNA into double-stranded DNA before gene expression can be initiated. Strategies developed to overcome this limitation include the construction of double-stranded DNA vectors, but this results in further reduction in the size capacity of transgene insertion, as one has to incorporate the gene twice into the vector (in its sense and antisense orientation). (3) A possible association between AAV2 vector gene transfer and tumorigenesis has been suggested, in a preclinical study with an animal model for mucopolysaccharidosis VII (MPS VII) [16]. To date, these results have not yet been reproduced and the cause for the malignancies is still unclear. And (4), some investigators have shown preferential integration of recombinant AAV2 vectors into transcriptionally active chromatin regions [17,18]. Nevertheless, the overall frequency of rAAV2 integration is very low and it is not clear yet whether this is a general phenomenon or specific for liver or the model used or AAV2.

### The state of the art of AAV-based vectors in gene therapy

**Figure 1:**
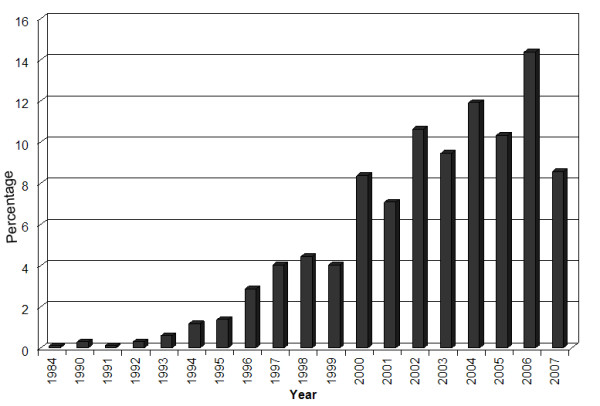
Percentage of published papers on AAV gene therapy, according to PubMed search (until June 2007).

Despite existing limitations and troubles to be resolved and overcome, rAAV-based gene transfer vectors still represent one of the most promising gene therapy systems and gain increasing popularity. In a search for "AAV gene therapy", 1,016 PubMed references were recovered (until June 2007). The first paper, on the use of AAV as mammalian DNA cloning vector, was published in 1984 by Hermonat and Muzyczka [9]. Since then, a rapidly growing number of studies on AAV-based vectors have been published, as shown in Figure 1.

The initial studies described aspects of the virology and molecular biology of the virus, virus isolation, as well as methods for gene transfer and expression in *in vitro *studies. As this vector system was used with increasing in gene delivery protocols, the interest in the complexities of AAV biology and transduction ability has also raised, and a considerable diverse potential for different cell types and target tissues was described [19]. Simultaneously, research efforts concentrated on rAAV construction, production and purification, as well as on the understanding and improvement of their functioning.

**Figure 2:**
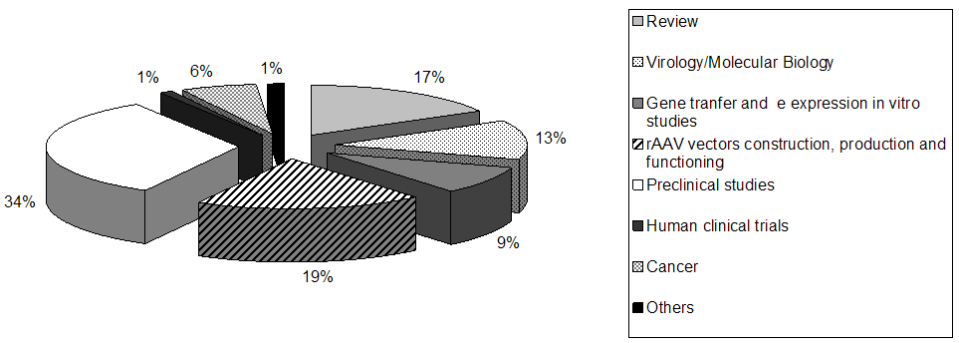
Publications on AAV-based gene therapy per area.

**Figure 3:**
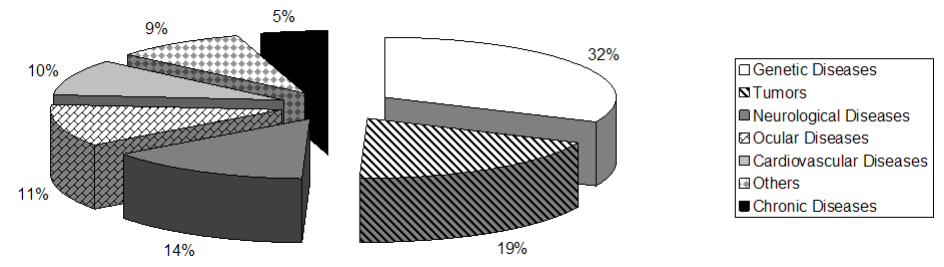
Target diseases for AAV gene therapy approaches.

Publications on the use of AAV-based vectors in gene therapy may be classified into ten large groups (Figure 2): (1) review; (2) virology and molecular biology; (3) gene transfer and expression studies *in vitro*; (4) construction, production and functioning of rAAV vectors; (5) preclinical studies; (6) human clinical trials; (7) preclinical and clinical *ex-vivo *approaches; (8) cancer; (9) vector biodistribution and routes of vector administration; (10) association of cellular and gene therapy. As presented in Figure 3, the great majority of papers concerns to preclinical studies, followed by studies on the construction, production and functioning of rAAV vectors. Human clinical trials are just beginning to appear in this scenario.

If each area is analyzed along the time, we can observe that studies concerning AAV virology and molecular biology are relatively constant, which is to be expected since basic research is of crucial importance to provide the information needed for pre-clinical and clinical studies. *In vitro *studies have been decreasing in number, to the advantage of *in vivo *and preclinical studies which have had their peak in the least year. Pre-clinical studies and clinical trials using AAV-based vectors will be detailed below.

The analysis of publications available in the PubMed show that several animals have been used in preclinical studies investigating the use of AAV-based. The mouse is the most frequently used animal, corresponding to about 68% of preclinical studies. The second most frequent animal model is the rat, used in 19% of the studies. Primate and canine models are used in 10% and 13% of *in vivo *animal studies, respectively. Other less expressive models are the guinea pig, rabbits, hamster (5% each one) and gerbil (only one study).

Recombinant AAV2 vectors have been tested in preclinical studies for a variety of diseases such as hemophilia, ∝1-anti-trypsin deficiency, cystic fibrosis, Duchenne muscular dystrophy, rheumatoid arthritis and others. Figure 2 presents the type and frequency of target diseases for which AAV-based vectors gene therapy is under study. Genetic diseases is the leading group of target diseases (32%), followed by tumors (19%), neurological disorders (14%), ocular (11%) and cardiovascular (10%) diseases, chronic disorders (5%) and others (9%). For all of them, the majority of papers concerns to both preclinical and clinical *in vivo *studies, although clinical trials are still scarce. In a review published in 2004 about the use of AAV vectors for the treatment of inherited disorders [19], hemophilia was shown as the major target disease, corresponding to about 37% of the published papers. Muscular dystrophy, cystic fibrosis and lysosomal storage disorders contributed with 20%, 18% and 20% of all reports, respectively. Other genetic diseases are still poorly investigated regarding AAV-based gene therapy approaches.

In our PubMed research, we found 15 publications describing the results of clinical trials. Among them, 13 are phase I trials and two are phase II studies. Currently, several clinical trials evaluate the use of AAV vectors for genetic and acquired diseases [20-22]. From 1989 until now, a total of 1,283 gene therapy clinical trials have been approved. Only 4% (47) of them are AAV-based gene therapy trials, distributed in phase I (66%), phase I/II (17%), phase II (6%) and phase III (11%). The first trial was approved in 1994, but most of the trials were approved in 2004, 2005 and 2006 [22]. Table 1 shows the number of clinical trials with AAV-based vectors and their status in June 2007.

**Table 1:** Clinical trials with AAV-based vectors [22].

**Category/Disease**	**Clinical trial**				
	
	Phase I O/C*	**Phase I/II O/C**	**Phase II O/C**	**Phase III O/C**	**Total**
**Infeccious**					
HIV vaccine	2/0	0/0	0/0	0/0	2

**Cancer**					
Malignant melanoma	1/0	0/0	0/0	0/0	1
Prostate cancer	0/1	2/1	0/0	4/0	8
Hormone refractory prostate cancer	1/0	0/0	0/0	0/0	1
Metastatic prostate cancer	0/0	0/0	0/0	1/0	1

**Genetic**					
Lipoprotein lipase deficience	0/0	1/0	0/0	0/0	1
Haemophilia B	1/2	0/0	0/0	0/0	3
Early onset retinal degeneration	3/0	0/0	0/0	0/0	3
Cystic fibrosis	2/3	1/1	0/2	0/0	9
Muscular dystrophy	1/0	0/0	0/0	0/0	1
Canavan disease	1/0	0/0	0/0	0/0	1
Duchenne muscular dystrophy	1/1	0/0	0/0	0/0	2
Limb Girdle muscular dystrophy	1/0	0/0	0/0	0/0	1
Amyotrophic lateral sclerosis	1**/0	0/0	0/0	0/0	1
Inherited autosomal recessive alpha-1-antitrypsin deficiency	2/0	0/0	0/0	0/0	2
Late infantile neuronal ceroid lipofuscinosis	1/0	0/0	0/0	0/0	1

**Neurological**					
Parkinson's disease	1/1	0/0	1/0	0/0	3
Alzheimer's disease	0/0	0/1	0/0	0/0	1
Epilepsy	1/0	0/0	0/0	0/0	1

**Cardiovascular**					
Heart failure	2/0	0/0	0/0	0/0	2

**Others**					
Rheumatoid arthritis	2/0	0/0	0/0	0/0	2

**TOTAL**	24/8	4/3	1/2	5/0	47

* O/C, open/closed; ** under review.

Despite the fact that the main target disease for gene therapy clinical trial is cancer (67%), followed by cardiovascular disease (9.1%) and monogenic diseases (8.4%), AAV-based gene therapy trials are mainly focused on monogenic diseases (53%), followed by cancer that corresponds to 23% [22].

The first clinical trial results were published in 1999, with the treatment of cystic fibrosis patients with an AAV-based vector [23]. Currently, this is the disease most frequently treated with AAV vectors [24-26], followed by hemophilia B [27,28]. There are also phase I human trials for ∝1-anti-trypsin deficiency [29], Canavan disease [30-32], infantile neuronal ceroid lipofuscinosis [33] and Parkinson disease [34]. The only two phase II clinical trials already published were directed to cystic fibrosis [35,36]. At least 20 clinical trials have been completed or initiated with 15 different AAV2-based vectors being administered in several hundred patients [37].

## Results and perspectives

The studies so far have shown that AAV-based vectors, and particularly the AAV2 serotype, are in general safe and efficient tools for gene transfer, but have also pointed out that transduction efficiency of AAV2 vectors falls short of requirements for adequate and organ-specific transgene expression. As a result, research efforts focused on modifying both vector genomes and capsid proteins to improve the transduction efficiency and/or specificity of AAV2-based vectors have been emerging. Self-complementary AAV2 vectors [38-40], for instance, were developed to bypass rate-limiting second-strand DNA synthesis and display enhanced transduction in comparison with conventional AAV vectors in some organs and tissues as liver [38-40], muscle [40], brain [41], retina [42] and cancer cells [43]. Other efforts have focused on manipulating the AAV2 capsid through site-directed and insertional mutagenesis, peptide display libraries, and chemical conjugation [44,45].

The repertoire of rAAV vectors has been greatly expanded by the development of technologies to pseudo-package rAAV genomes, package AAV genomes with two different ITR serotypes, generate mosaic rAAV particles with more than one capsid serotype, retarget AAV by generating rAAV capsid modification and generate rAAV with chemically modified capsids. These technologies have greatly expanded the ability to fit rAAV for specific gene therapy applications [46,47].

These publications reveal also a great interest on rAAV biology, concerning particularly virus intracellular trafficking which has been shown a major rate-limiting step in rAAV transduction for many cell types. Moreover, it has also been indicated as critically affecting host immunological response toward input capsids in the absence of new viral protein synthesis. If so, altering the rate of intracellular trafficking and uncoating of rAAVs by the use of specific drugs or serotype modifications could directly influence the stability of gene expression, by reducing host immune responses that promote the clearance of virus-infected cells. Advances in understanding rAAV biology will lead to the improvement of the efficacy of this vector system for the treatment of inherited and acquired diseases.

## Conclusion

Progress in gene therapy is indisputable but has been slow and there have been many ups and downs. The two major hurdles to gene therapy, safety and efficacy, remain roadblocks to the widespread application of gene therapy as a standard medical treatment for disease. Improvement of efficacy can be mediated in part by the development of more efficient vectors. Retrovirus-based vectors represented the first attempt to use viral vectors, and were considered the great promise for gene therapy approaches. However, after the serious adverse events occurred with the Moloney virus, retrovirus had their potential questioned. Later, the development of lentiviral-based vectors renewed gene therapy expectations. Currently, although these vectors have been shown in preclinical studies to mediate high levels of stable gene transfer for long-term expression, there is reasonable concern regarding important safety aspects, in particular regarding recombination of a lentiviral vector into a replication-competent lentivirus (RCL) that might represent a novel and unpredictable pathogen; and insertional oncogenesis mediated by the "random" insertion of retroviruses into cellular DNA [48]. Presently, other virus vectors have been gaining an important place in gene therapy approaches, especially adenovirus and adeno-associated virus.

Since the adeno-associated virus was first isolated and its biological properties established, it has been considered a promising vector for gene therapy. Research approaches are disclosing advantages of this tools. Some obstacles have already been overcome, others are rising and need to be surpassed, and research advances will certainly bring more challenges for the near future. Nevertheless, AAV-based vectors seem to bypass the main gene therapy barriers, such as long-term and stable transgene expression in many tissues, safety, broad range of target diseases and lack of immunogenicity and pathogenicity.

## Competing interests

The author(s) declare that they have no competing interests.

## Authors' contributions

RSC was mainly involved with the PubMed research. RSC and NBN contributed equally to the writing of the manuscript.
