# Correction: The state of the art of adeno-associated virus-based vectors in gene therapy

**DOI:** 10.1186/1743-422X-7-12

**Published:** 2010-01-21

**Authors:** Renata dos Santos Coura, Nance Beyer Nardi

**Affiliations:** 1 Department of Genetics, Universidade Federal do Rio Grande do Sul, Av Bento Goncalves 9500, 91501-970, Porto Alegre, RS, Brazil

## Abstract

It has come to our attention that, on writing our manuscript (dos Santos Coura and Nardi, 2007), we unintentionally included unquoted passages from the work by Wu et al. (2006), collected during the period when we were doing extensive readings on the subject, and not adequately referenced. We regret this error and offer our sincere apologies.

## Correction

It has come to our attention that, on writing our manuscript [1], we unintentionally included unquoted passages from the work by Wu et al. (2006) [2], collected during the period when we were doing extensive readings on the subject, and not adequately referenced. We regret this error and offer our sincere apologies.
